# Disrupted Spontaneous Neural Activity Related to Cognitive Impairment in Postpartum Women

**DOI:** 10.3389/fpsyg.2018.00624

**Published:** 2018-05-03

**Authors:** Jin-Xia Zheng, Yu-Chen Chen, Huiyou Chen, Liang Jiang, Fan Bo, Yuan Feng, Wen-Wei Tang, Xindao Yin, Jian-Ping Gu

**Affiliations:** ^1^Department of Radiology, Nanjing First Hospital, Nanjing Medical University, Nanjing, China; ^2^Department of Radiology, The Affiliated Obstetrics and Gynecology Hospital of Nanjing Medical University, Nanjing Maternity and Child Health Care Hospital, Nanjing, China

**Keywords:** postpartum women, ALFF, ReHo, resting-state fMRI, spontaneous activity

## Abstract

**Purpose:** Prior research has demonstrated that the postpartum period is associated with an increased risk of cognitive impairment. This study aims to investigate whether disrupted spontaneous neural activity exists in postpartum women without depression using resting-state functional magnetic resonance imaging (rs-fMRI) and to detect the relationship between these abnormalities and cognitive impairment.

**Materials and Methods:** Postpartum women (*n* = 22) were compared with age- and education-matched nulliparous women (*n* = 23) using rs-fMRI. We calculated the amplitude of low-frequency fluctuation (ALFF) and regional homogeneity (ReHo) values to evaluate spontaneous neural activity and detect the relationship between rs-fMRI data and cognitive performance.

**Results:** Relative to nulliparous women, postpartum women had significantly decreased ALFF and ReHo values primarily in the left posterior cingulate cortex (PCC) and prefrontal cortex and increased ALFF values in left cerebellar posterior lobe. We found a positive correlation between the ALFF and ReHo values in the PCC and the complex figure test (CFT)-delayed scores in postpartum women (*r* = 0.693, *p* = 0.001; *r* = 0.569, *p* = 0.011, respectively). Moreover, the clock-drawing test (CDT) scores showed positive correlations with the ALFF and ReHo values in the right superior frontal gyrus (SFG; *r* = 0.492, *p* = 0.033; *r* = 0.517, *p* = 0.023, respectively).

**Conclusion:** Our combined ALFF and ReHo analyses revealed decreased spontaneous neural activity, mainly in the PCC and prefrontal cortex, which was correlated with specific impaired cognitive functioning in postpartum women. This study may elucidate the neurophysiological mechanisms underlying postpartum cognitive impairment and enhance our understanding of the neurobiological aspects of the postpartum period.

## Introduction

Postpartum women experience a multitude of physical and environmental changes and are at risk of developing worsening of underlying affective disorders (Munk-Olsen et al., 2009, 2012; Agrati and Lonstein, 2016; Esscher et al., 2016; Gingnell et al., 2017). Furthermore, the postpartum period has been linked with an increased risk of cognitive impairment, which primarily presents as poor memory or recent memory loss, forgetfulness, difficulty concentrating, and distractibility (Christensen et al., 2010; Postma et al., 2014; Albin-Brooks et al., 2017). Alterations in hormone levels, such as estrogen, progesterone, and glucocorticoid levels, may result in cognitive impairment during the postpartum period (Henry and Sherwin, 2012). Previous studies have indicated that women during the postpartum period have significant cognitive deficits that may occur prior to affective disorder (Christensen et al., 2010; Postma et al., 2014; Meena et al., 2016). Postpartum-related cognitive impairment may play a major role in various postpartum psychiatric disorders (Henry and Rendell, 2007; Chan et al., 2015; Hoekzema et al., 2017). However, most studies are focusing on the postpartum associated affective disorders such as depression and anxiety, while few studies have compared the cognitive function and behavioral problems in postpartum women without affective disorders. Moreover, the neuropathological mechanism of postpartum cognitive impairment still remains largely unknown.

Neuroimaging techniques have been applied to investigate the anatomical and functional alterations in the brain in postpartum women. Gray matter (GM) atrophy and white matter (WM) lesions, which are common structural abnormalities that have been observed in previous studies, are modestly linked with postpartum cognitive decline (Kim et al., 2010; Postma et al., 2014; Hoekzema et al., 2017). Hoekzema et al. (2017) demonstrated that primiparous women underwent a symmetrical pattern of extensive GM volume reductions throughout pregnancy, which primarily affected the anterior and posterior cortical midline and bilateral lateral prefrontal and temporal cortex. The GM reductions endured for at least 2 years post-pregnancy (Hoekzema et al., 2017). Nevertheless, little is known concerning the complex neurophysiological activity in the central nervous system of postpartum women. Neural abnormalities have been detected in populations at risk for developing cognitive impairment (Machulda et al., 2011). Therefore, measures of neural activity could conceivably be used to detect and track the potential effects of postpartum cognitive impairment on brain function.

Prior studies have used task-based functional magnetic resonance imaging (fMRI) to examine the effects of emotion reactivity on brain activity during the postpartum period (Gingnell et al., 2015, 2017). Resting-state fMRI (rs-fMRI) is a powerful tool for evaluating spontaneous neural activity (Mantini et al., 2007; Lee et al., 2013) and rs-fMRI has been used to investigate the disrupted functional connectivity network in postpartum depressed women (Xiao-juan et al., 2011; Chase et al., 2013; Deligiannidis et al., 2013; Fisher et al., 2016). However, very few studies have investigated the spontaneous brain activity in postpartum women without depression and its effects on cognitive function.

The amplitude of low-frequency fluctuation (ALFF) and regional homogeneity (ReHo) are two major data-driven algorithms for local measure of spontaneous neural activity. ALFF measures the amplitude of very low-frequency oscillations of the blood oxygenation level-dependent (BOLD) signal at the single-voxel level (Zang et al., 2007), whereas ReHo analyzes the neural synchronization of a given voxel with its adjacent voxels, i.e., local neural synchrony (Zang et al., 2004). The combination of ALFF and ReHo may provide a more comprehensive pathophysiological assessment of human brain dysfunction than either method alone. Therefore, we took advantage of the two most common methods for whole-brain analyses to identify the spontaneous neural activity in postpartum women.

On the basis of prior work and theoretical considerations, we aimed to combine ALFF and ReHo to explore the spontaneous neural activity in postpartum women compared with that in nulliparous women and hypothesized that (1) aberrant ALFF and ReHo values would be identified within several brain regions involved in emotional processing, attention, and cognitive function and (2) disrupted spontaneous neural activity would be associated with impaired cognitive performance.

## Materials and Methods

### Subjects

This study was approved by the Research Ethics Committee of the Nanjing Medical University. All individuals provided written informed consent before their participation in the study protocol (Reference No. 2016067).

In this study, a total of 46 subjects (aged between 20 and 40 years, all right-handed with the completion of at least 9 years of education) made up of 23 postpartum women and 23 nulliparous women were included through community health screening and newspaper advertisements. One postpartum woman was subsequently excluded because of the exceeded limits for head motion during scanning. All the postpartum women were primiparous and medication free and had delivered a healthy and full-term infant in the preceding 3 months. None of the women experienced any complications during pregnancy or delivery, such as hypertension, diabetes, eclampsia, heart disease, or postpartum hemorrhage. Among them, 11 women had natural childbirth and the other 11 chose caesarean section. Sixteen women were breastfeeding and the other six women were mix-feeding.

Eight of the women carried a boy and 12 a girl. The remaining 2 had twins (1 had male twins and 1 had female twins). Considering the previously reported effects of fetal sex on cognitive changes in pregnant women (Vanston and Watson, 2005), we additionally compared the women carrying a boy to the women carrying a girl (excluding the two women having twins). No differences in structural and functional alterations were observed between the two groups.

Women were excluded from the study if they had severe smoking, alcoholism, stroke, brain trauma, Parkinson’s disease, Alzheimer’s disease, major depression, epilepsy, neuropsychic disorders that could affect cognitive function, major medical illness (e.g., anemia, thyroid dysfunction, and cancer), MRI contraindications, or were currently pregnant. None of the postpartum women had symptoms of postnatal depression according to the Edinburgh Postnatal Depression Scale (EPDS, overall scores < 12; Cox et al., 1987). Moreover, none of the included participants had accompanying symptoms including depression and anxiety, according to the Self-Rating Depression Scale (SDS) and Self-Rating Anxiety Scale (SAS; overall scores < 50, respectively; Zung, 1971, 1986). The characteristics of the postpartum women and nulliparous women are summarized in **Table 1**.

**Table 1:** Demographics, clinical, and cognitive characteristics of the postpartum women and nulliparous women.

	Postpartum women (*n* = 22)	Nulliparous women (*n* = 23)	*p* value
Age (year)	29.32 ± 2.93	29.43 ± 3.87	0.910
Education levels (years)	17.09 ± 1.77	17.35 ± 3.04	0.732
Fasting glucose (mmol/L)	4.85 ± 0.47	4.81 ± 0.35	0.795
Triglycerides (mmol/L)	4.45 ± 0.74	4.14 ± 0.58	0.122
Total cholesterin (mmol/L)	0.99 ± 0.40	0.80 ± 0.30	0.073
LDL-cholesterin (mmol/L)	2.54 ± 0.62	2.34 ± 0.48	0.240
HDL-cholesterin (mmol/L)	1.48 ± 0.33	1.38 ± 0.24	0.244
WM hyperintensity	0 (0–1)	0 (0–2)	0.637
FD value	0.21 ± 0.06	0.20 ± 0.07	0.904
**Cognitive performance**			
MMSE	28.95 ± 0.95	29.22 ± 0.80	0.319
MoCA	26.14 ± 1.17	26.30 ± 1.11	0.622
AVLT	34.27 ± 8.78	33.78 ± 7.37	0.840
AVLT-delayed recall	7.27 ± 2.19	6.52 ± 1.88	0.223
CFT	34.68 ± 1.57	34.41 ± 1.87	0.605
CFT-delayed recall	16.45 ± 3.11	18.22 ± 1.44	0.022*
DST	15.00 ± 3.24	13.74 ± 2.91	0.176
TMT-A	44.09 ± 9.20	48.70 ± 9.62	0.108
TMT-B	83.59 ± 15.18	90.26 ± 10.66	0.098
CDT	3.18 ± 0.59	3.57 ± 0.51	0.024*
VFT	15.55 ± 3.40	14.46 ± 3.11	0.269
DSST	69.82 ± 9.72	69.70 ± 8.08	0.963
EPDS	4.18 ± 2.52	–	–
SDS	38.50 ± 6.15	38.09 ± 5.88	0.819
SAS	40.23 ± 7.35	38.78 ± 7.08	0.505

*Data are represented as mean ± SD or median (range). ^∗^*p* value < 0.05. LDL, low-density lipoprotein; HDL, high-density lipoprotein; WM, white matter; FD, framewise displacement; MMSE, Mini Mental State Exam; MoCA, Montreal cognitive assessment; AVLT, auditory verbal learning test; CFT, complex figure test; DST, digit span test; TMT-A, trail-making test – Part A; TMT-B, trail-making test – Part B; CDT, clock-drawing test; VFT, verbal fluency test; DSST, digit symbol substitution test; EPDS, Edinburgh Postnatal Depression Scale; SDS, Self-Rating Depression Scale; SAS, Self-Rating Anxiety Scale.*

### Clinical Data and Neuropsychological Assessment

To exclude the hyperglycemia and hyperlipidemia at the time of examination, blood samples of all of the participants were collected after an 8-h fast by venipuncture at 8 A.M. to assess the levels of fasting plasma glucose (FPG), triglycerides, total cholesterol, low-density lipoprotein (LDL)-cholesterol, and high-density lipoprotein (HDL)-cholesterol.

All subjects underwent a battery of neuropsychological tests that covered related cognitive domains. The neuropsychological status of the participants was established using the Mini Mental State Exam (MMSE; Galea and Woodward, 2005), Montreal cognitive assessment (MoCA; Nasreddine et al., 2005), auditory verbal learning test (AVLT; Schmidt, 1996), complex figure test (CFT; Shin et al., 2006), digit span test (DST; Hale et al., 2002), trail-making test (TMT) A and B (Bowie and Harvey, 2006), clock-drawing test (CDT; Samton et al., 2005), verbal fluency test (VFT; Brucki and Rocha, 2004), and digit symbol substitution test (DSST; Bettcher et al., 2011). The tests assessed general cognitive function, episodic verbal and visual memory, semantic memory, attention, psychomotor speed, executive function, and visuospatial skills. It took approximately 60 min for each individual to complete all of the tests in a fixed order.

### MRI Data Acquisition

All subjects were scanned using a 3.0 Tesla MRI scanner (Ingenia, Philips Medical Systems, Netherlands) with an eight-channel receiver array head coil. Head motion and scanner noise were alleviated using foam padding and earplugs. The earplugs (Hearos Ultimate Softness Series, United States) were used to attenuate scanner noise by approximately 32 dB based on the manufacture’s data sheet. Subjects were instructed to lie quietly with their eyes closed and without falling asleep, not to think about anything in particular, and to avoid any head motion during the scan. Functional images were acquired axially using a gradient echo-planar imaging sequence as follows: repetition time (TR), 2000 ms; echo time (TE), 30 ms; slices, 36; thickness, 4 mm; gap, 0 mm; flip angle (FA), 90°; field of view (FOV), 240 mm × 240 mm; and acquisition matrix, 64 × 64. Structural images were acquired with a three-dimensional turbo fast echo (3D-TFE) T1WI sequence with high resolution as follows: TR/TE, 8.2/3.8 ms; slices, 170; thickness, 1 mm; gap, 0 mm; FA, 8°; acquisition matrix, 256 × 256; and FOV, 256 mm × 256 mm. Fluid-attenuated inversion recovery (FLAIR) scans were also acquired: TR/TE, 7000/120 ms; slices, 18; thickness, 6 mm; gap, 1.3 mm; FA, 110°; and voxel size, 0.65 mm × 0.95 mm × 6 mm.

### Functional Data Preprocessing

Functional data analyses were conducted using the Data Processing Assistant for Resting-State fMRI (DPARSF) programs (Yan and Zang, 2010) based on statistical parametric mapping (SPM8^1^) and the rs-fMRI data analyses toolkits (REST^2^). The first 10 volumes were removed from each time series to account for the time it took participants to adapt to the scanning environment. Slice timing and realignment for head-motion correction were then performed for the remaining 230 images. Participant data exhibiting head motion >2.0 mm translation or >2.0° rotation were excluded from analysis. The remaining dataset was spatially normalized to the Montreal Neurological Institute template (resampling voxel size = 3 mm × 3 mm × 3 mm). Qualified images were processed using a linear trend and band-pass filtering (0.01–0.08 Hz). Any subjects with a head motion >2.0 mm translation or a 2.0° rotation in any direction were excluded.

### ALFF and ReHo Analyses

For ALFF analysis, the images were first smoothed with a Gaussian kernel of 8 mm full-width at half-maximum (FWHM). Next, the time series were first transformed to the frequency domain using a fast Fourier transform. The square root was then computed at each frequency of the power spectrum. ALFF values were acquired after the calculation at each frequency of the power spectrum across 0.01–0.08 Hz at each voxel. For standardization purposes, the ALFF of each voxel was divided using the global mean ALFF value. ALFF was finally calculated using the REST software through the procedure described in previous studies (Zang et al., 2007).

For ReHo analysis, the images were analyzed to calculate Kendall’s coefficient of concordance of the time courses of a given voxel with its 26 nearest neighboring voxels. The ReHo maps were then spatially smoothed with a Gaussian kernel of 6 mm. The ReHo value of each voxel was standardized by dividing the raw value by the global mean ReHo value that was acquired with a computation similar to that used to calculate the global mean ALFF value. ReHo was also calculated using the REST software through the procedure described in previous studies (Zang et al., 2004).

### Structural Analysis

Structural images of each subject were processed using the VBM toolbox software in SPM8^3^. Briefly, the structural images were normalized and segmented into GM, WM, and cerebrospinal fluid (CSF) using the unified segmentation model in SPM8 (Ashburner and Friston, 2005). The brain parenchyma volume was calculated as the sum of the GM and WM volumes. The GM, WM, and brain parenchyma volume were divided by the total intracranial volumes to adjust for variability due to head size. The GM images were spatially smoothed using a Gaussian kernel of 8 mm FWHM. The voxel-wise GM volume was used in the following statistical analysis as covariates for the ALFF and ReHo calculations.

Since the presence of the WM lesions may be an important risk marker for the development of cognitive impairment (de Groot et al., 2000, 2001), quantitative assessment of WM hyperintensity was performed on the FLAIR images using the age-related WM changes scale (Wahlund et al., 2001) by two experienced radiologists (XY and J-PG) who were blinded to the clinical data and group allocation. Participants with a rating score above 1 (confluence of lesions or diffuse involvement of the entire region) were excluded. Consensus was obtained through a discussion between the two assessors.

### Statistical Analysis

Demographic variables and cognitive performance scores were compared between both groups using SPSS 20.0 (SPSS, Inc., Chicago, IL, United States). An independent two-sample *t*-test was used for continuous variables, and a χ^2^ test was applied for proportions. Values of *p* < 0.05 were considered statistically significant.

#### Within-Group Analysis

For within-group analysis, one-sample *t*-tests were performed on the individual ALFF and ReHo maps in a voxel-wise manner for postpartum women and nulliparous women. Significant thresholds were corrected using false discovery rate (FDR) criterion and set at *p* < 0.001.

#### Between-Group Analysis

For between-group analysis, two-sample *t*-tests were performed to investigate the differences of ALFF and ReHo values between postpartum women and nulliparous women using a default GM mask. Age and education were included as null covariates. To exclude the potential effects of GM volume, the voxel-wise GM volume maps were also included as covariates. Multiple comparison correction was performed using FDR criterion, and the significance was set at *p* < 0.001 according to the suggestion from a prior study (Eklund et al., 2016).

#### Correlation Analysis

To investigate the relationship between regional ALFF and ReHo values, a bivariate correlation was performed between these two algorithms. Briefly, the mean ALFF and ReHo values with significant differences were individually extracted and correlated with one another.

To investigate the relationship among the ALFF/ReHo values of the peak voxels and neuropsychological performance, Pearson’s correlation analyses were performed using SPSS software since data distributions meet the requirements of parametric statistics. Partial correlations were analyzed using the age, education, and GM volume as covariates. A value of *p* < 0.05 was considered to be statistically significant. Bonferroni correction was used for multiple comparisons in the correlation analyses. The Bonferroni corrected *p* values for ALFF and ReHo were 0.00083 and 0.00139, respectively.

Since micromovements from volume to volume can influence spontaneous neuronal activity (Power et al., 2012), framewise displacement (FD) values were computed for each subject to reflect the temporal derivative of the movement parameters. No subjects had FD > 0.5 mm for more than 35 volumes in this study. No significant difference was found in the mean FD values between postpartum women and nulliparous women (**Table 1**).

## Results

### Demographic and Neuropsychological Characteristics

**Table 1** summarizes the demographic measures and neuropsychological test results of the postpartum women and the nulliparous women. The two groups did not significantly differ in terms of age, education level, FPG, blood lipids, and WM hyperintensity (all *p* > 0.05). In terms of the cognitive assessment, the postpartum women had significantly poorer CFT-delay and CDT scores than the nulliparous women (all *p* < 0.05). The other neuropsychological tests showed no significant differences between postpartum women and nulliparous women.

### Structural Analysis

**Table 2** shows no significant difference in GM, WM, or brain parenchyma volume between postpartum women and nulliparous women. After Monte Carlo simulation correction, no suprathreshold voxel-wise difference in the GM and WM volume between the postpartum women and nulliparous women was observed. None of the participants in this study were excluded due to severe atrophy.

**Table 2:** Comparisons of the brain volumes between postpartum women and nulliparous women.

	Postpartum women (*n* = 22)	Nulliparous women (*n* = 23)	*p* value
Gray matter volume (% of TIV)	31.6 ± 1.8	32.2 ± 1.3	0.146
White matter volume (% of TIV)	29.3 ± 1.4	29.5 ± 1.5	0.720
Brain parenchyma volume (% of TIV)	60.9 ± 2.9	61.7 ± 2.3	0.285

*Data are expressed as mean ± SD. TIV, total intracranial volume.*

### ALFF and ReHo Analysis

**FIGURE 1:**
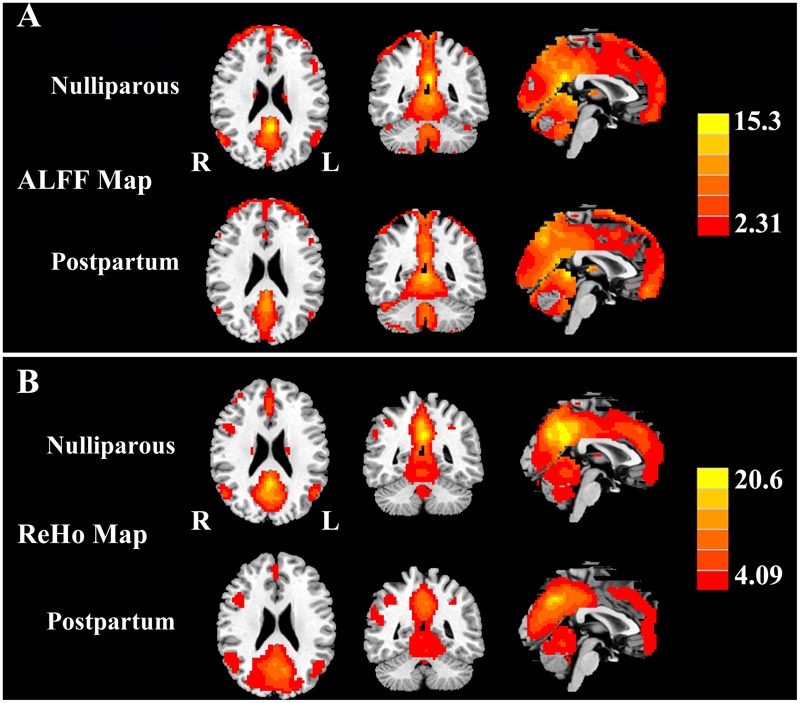
**(A)** One-sample *t*-test results of ALFF maps (*p* < 0.001 corrected by FDR) in nulliparous women and postpartum women. **(B)** One-sample *t*-test results of ReHo maps (*p* < 0.001 corrected by FDR) in nulliparous women and postpartum women. The left side corresponds to the right hemisphere.

**FIGURE 2:**
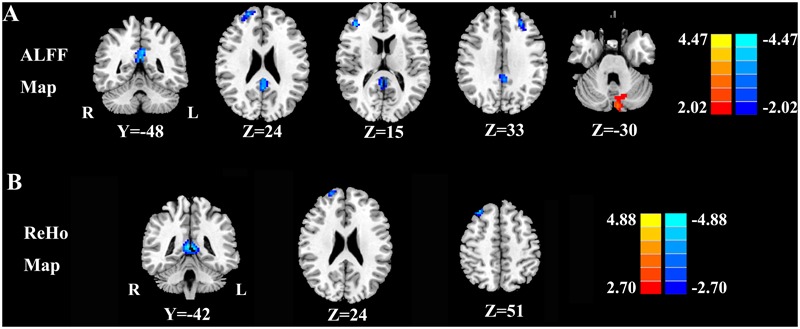
**(A)** Regions exhibiting differences in ALFF between the nulliparous women and postpartum women (*p* < 0.001 corrected by FDR). **(B)** Regions exhibiting differences in ReHo between nulliparous women and postpartum women (*p* < 0.001 corrected by FDR). The left side corresponds to the right hemisphere.

**Figure 1** shows the standardized ALFF (**Figure 1A**) and ReHo (**Figure 1B**) heat maps for the nulliparous women and postpartum women. The ALFF and ReHo values were significantly greater than the global mean values mainly in the posterior cingulate cortex (PCC), superior frontal gyrus (SFG), middle frontal gyrus (MFG), inferior parietal lobe (IPL), and cerebellum. Compared with the nulliparous women, the postpartum women had significantly decreased ALFF values in the left PCC, right SFG, and bilateral MFG but increased ALFF values in the left cerebellar posterior lobe (CPL; **Figure 2A** and **Table 3**). Compared to the nulliparous women, the postpartum women had significantly lower ReHo values in the left PCC, right SFG, and right MFG, but no significant increases were observed (**Figure 2B** and **Table 3**). Furthermore, we did not observe any significant differences in cognitive functions and resting-state neural activity between women with natural childbirth and women with caesarean section (all *p* > 0.05).

**Table 3:** Regions showing significant differences in ALFF and ReHo values between postpartum women and nulliparous women.

Brain regions	BA	Peak MNI coordinates *x, y, z* (mm)	Peak *T* value	Voxels
ALFF differences				
PCC	31	-3, -48, 30	-3.8499	135
R SFG	10	21, 57, 24	-3.6502	47
R MFG	10	42, 42, 15	-3.7782	54
L MFG	9	-24, 51, 33	-3.7039	58
L CPL	–	-6, -84, -30	3.3354	65
ReHo differences				
PCC	31	3, -42, 9	-4.3286	125
R SFG	10	21, 60, 24	-3.6324	43
R MFG	8	30, 33, 51	-4.0864	61

*The threshold was set at a p < 0.001 (FDR corrected). BA, Brodmann’s area; MNI, Montreal Neurological Institute; PCC, posterior cingulate cortex; SFG, superior frontal gyrus; MFG, middle frontal gyrus; CPL, cerebellar posterior lobe; L, left; R, right.*

### Correlation Analysis

**FIGURE 3:**
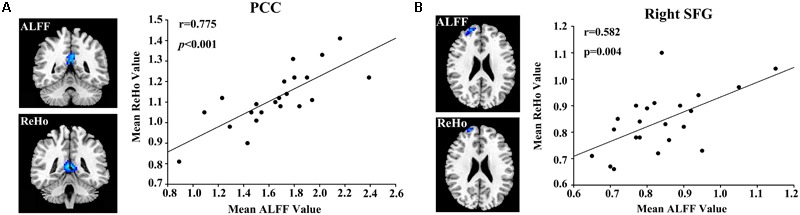
Correlations between the ALFF and ReHo values in the **(A)** posterior cingulate cortex (PCC; *r* = 0.775, *p* < 0.001) and **(B)** right superior frontal gyrus (SFG; *r* = 0.582, *p* = 0.004).

**FIGURE 4:**
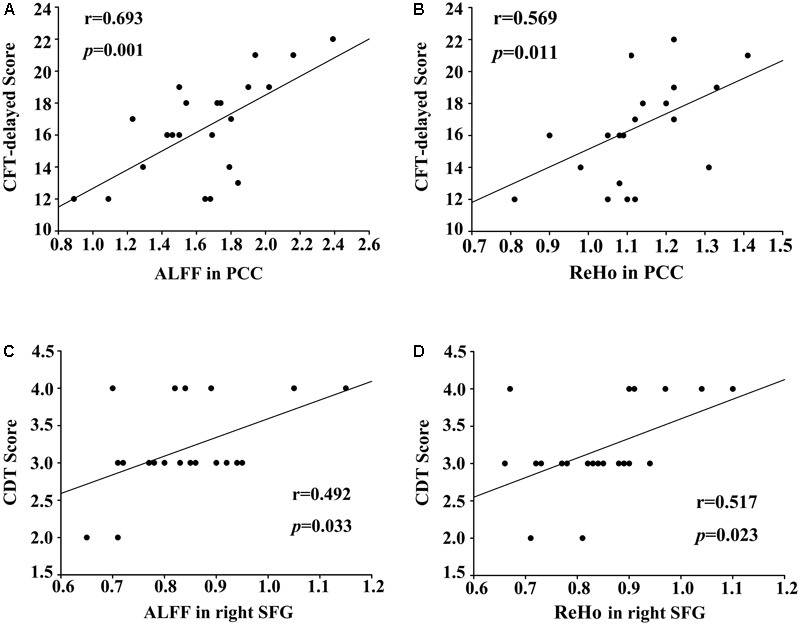
**(A)** Correlations between the CFT-delayed scores and decreased ALFF values in the PCC (*r* = 0.693, *p* = 0.001). **(B)** Correlations between the CFT-delayed scores and decreased ReHo values in the PCC (*r* = 0.569, *p* = 0.011). **(C)** Correlations between the CDT scores and reduced ALFF in the right SFG (*r* = 0.492, *p* = 0.033). **(D)** Correlations between the CDT scores and reduced ReHo in the right SFG (*r* = 0.517, *p* = 0.023).

The regional ALFF and ReHo values extracted from the PCC (*r* = 0.775, *p* < 0.001) and right SFG (*r* = 0.582, *p* = 0.004) were associated with each other after a bivariate correlation analysis (**Figure 3**). After a correction for age, education, and GM volume, we found a positive correlation between the ALFF and ReHo values in the PCC and the CFT-delayed scores in postpartum women (*r* = 0.693, *p* = 0.001; *r* = 0.569, *p* = 0.011, respectively; **Figure 4**). Moreover, the CDT scores showed positive correlations with the ALFF and ReHo values in the right SFG (*r* = 0.492, *p* = 0.033; *r* = 0.517, *p* = 0.023, respectively). However, no significant correlations persisted after Bonferroni correction, probably partly due to the relatively strict calculation. No such associations were detected in nulliparous women group. Additional decreases or increases in neural activity were independent of any other clinical characteristics and cognitive tests.

## Discussion

To our knowledge, this is for the first time to use both the ALFF and ReHo approaches to detect disrupted spontaneous neural activity related to cognitive impairment in postpartum women without depression. Decreased spontaneous neural activity was primarily detected in the PCC and prefrontal cortex. Moreover, significantly decreased neural activity in the PCC and SFG was strongly correlated with the impaired CFT-delayed and CDT scores found in postpartum women. These region-specific neural cognition associations may play a pivotal role in postpartum cognitive impairment.

Since pregnancy can lead to substantial changes in brain structure, we compared GM and WM volumes but did not detect any differences between postpartum women and nulliparous women. A previous study reported reduced GM volume in regions related to social cognition including the prefrontal and temporal cortices (Hoekzema et al., 2017). Moreover, Yeo et al. (2011) demonstrated the intersections between the GM volume changes of pregnancy and the cognitive components of the human association cortex. We speculate that the inherent heterogeneity of postpartum women may be one reason for the inconsistent results. Moreover, the MR technique and analytical method may also contribute to the differences. Given the lack of change in GM volume, our results suggest that altered spontaneous neural activity in postpartum women may occur prior to any major structural abnormalities.

ALFF and ReHo measurements have been widely used to explore the potential pathogenesis of various neuropsychiatric diseases, especially the cognition-related diseases (Zang et al., 2004, 2007; Wang et al., 2011; An et al., 2013; Cui et al., 2014; Han et al., 2015). These two analyses are based on different neurophysiological mechanisms with ALFF measuring the neural intensity (Zang et al., 2007) and ReHo representing the neural coherence (Zang et al., 2004). In the current study, decreased spontaneous neural activity was detected by both methods in the PCC and prefrontal cortex. Similarly, reduced ALFF and ReHo activities were also observed in the PCC and prefrontal cortex in patients with cognitive decline, such as Alzheimer’s disease (Wang et al., 2011), amnestic mild cognitive impairment (Zhang et al., 2012; Liang et al., 2014), and diabetes-related cognitive impairment (Cui et al., 2014). Thus, we suggest that these cognitive abnormalities may share the similar mechanisms with the cognitive changes in postpartum women. Moreover, the correlation between ALFF and ReHo values reflected the close relationship between both measurements. We propose that the coexisting neural intensity and coherence abnormalities in these specific regions may represent more severe brain changes than those revealed by a single method.

In our study, significant hypoactivity was found in default mode network (DMN) regions including the PCC, and was positively correlated with impaired CFT-delayed scores. The DMN, which consisted of nodes in the PCC/precuneus, bilateral IPL, medial temporal gyrus, and medial prefrontal gyrus, is most active at rest and shows reduced activity when a subject enters a task-based state involving attention or goal-directed behavior (Raichle et al., 2001; Mantini et al., 2007; Lee et al., 2013). As the central node of the DMN, the PCC performs diverse cognitive functions including visuospatial memory and processing of emotional and non-emotional information (Vogt et al., 2006; Leech and Sharp, 2013; Sestieri et al., 2017). Moreover, the CFT-delayed score was used to assess the visuospatial memory and visuospatial skills (Shin et al., 2006). Therefore, the correlation between decreased PCC activity and impaired CFT-delayed scores may indicate the decline of the visuospatial memory in postpartum women. The PCC is also recognized for its role in self-referential processing and social cognition (Mars et al., 2012). Johnson et al. (2006) confirmed that the anteroinferior PCC has more outward and preventative aspects of self-relevant thought, including duties and responsibilities to others. Postpartum women would be expected to have a stronger focus on infant-related responsibilities and be more involved in thinking about the intentions of others, especially the newborn (Chase et al., 2013). Furthermore, alterations in endogenous sex steroid hormone levels during the postpartum period may result in widespread neural changes, including in the PCC (Fisher et al., 2016). Thus, our results indicate that decreased ALFF and ReHo activity in the PCC may be responsible for the impaired visuospatial memory and self-referential processing in postpartum women.

The prefrontal cortex is mainly responsible for executive and cognitive functions (Yuan and Raz, 2014). In the current study, neural abnormalities in the prefrontal cortex were linked to impaired cognitive performance on CDT tests in postpartum women, which indicated the dysfunction of executive abilities as well as visuospatial processing and visuoconstructional skills. Using a neuropsychological assessment, previous studies have confirmed disrupted executive functioning as one of the main cognitive impairments in postpartum women (Anderson and Rutherford, 2012; Meena et al., 2016). The GM volume of the prefrontal cortex has been shown to be reduced after pregnancy (Hoekzema et al., 2017). In addition, decreased glutamatergic levels in the dorsolateral prefrontal cortex were observed in depressed postpartum women by proton magnetic resonance spectroscopy (MRS; Rosa et al., 2017). A study using fMRI showed that the prefrontal brain activity during a response inhibition task was decreased throughout the first postpartum weeks in healthy women (Bannbers et al., 2013). Our study extends the work of these aforementioned reports by showing that aberrant spontaneous activity in the prefrontal cortex may play a critical role in postpartum cognitive impairment, especially executive dysfunction.

By contrast, the left CPL showed enhanced ALFF values in postpartum women, which indicated the enhanced neural activity in specific regions that may be explained as a recruitment of additional neural resources to compensate for cognitive dysfunction in other brain regions (Reuter-Lorenz and Cappell, 2008; Sokolov et al., 2017). The association of the CPL with cognitive processing and emotion mediation has been proposed (Schmahmann and Caplan, 2006; Sokolov et al., 2017). It seems that hyperactivity in the CPL may be involved in limiting the cognitive decline during the postpartum period. However, since no correlations have been found between enhanced ALFF in the CPL and memory performance, the potential compensatory neural mechanism still requires further investigation.

Several limitations in this study must be acknowledged. First, this cross-sectional study involved a relatively small sample size, and we did not perform a follow-up fMRI comparison before and after pregnancy. Thus, it is difficult to make direct causal relationships between the disrupted spontaneous neural activity and cognitive impairment in postpartum women. Further longitudinal fMRI studies are required to establish and confirm the current findings. Second, there are currently no diagnostic criteria for postpartum cognitive impairment that may limit the interpretation of our results. Moreover, since brain WM volumes are not optimal for investigating WM tissue, the WM integrity needs to be evaluated using a more informative approach, such as diffusion tensor imaging (DTI). Finally, in addition to the spontaneous neural activity over the entire brain, functional connectivity measurements should be included to obtain more comprehensive information regarding different brain regions in postpartum women.

## Conclusion

In summary, our combined ALFF and ReHo analyses revealed decreased spontaneous neural activity, mainly in the PCC and prefrontal cortex, and this result was correlated with specific impaired cognitive functioning in postpartum women without depression. This study provides a new insight to investigate brain abnormalities and their relationships with cognitive impairment in postpartum women and enhances our understanding of the neurobiological aspects of the postpartum period.

## Author Contributions

J-XZ and Y-CC designed the experiment, collected the data, performed the analysis, and wrote the manuscript. HC, LJ, FB, and YF collected the data. W-WT, XY, and J-PG contributed to the discussion and manuscript revision.

## Conflict of Interest Statement

The authors declare that the research was conducted in the absence of any commercial or financial relationships that could be construed as a potential conflict of interest.

## Footnotes

1 http://www.fil.ion.ucl.ac.uk/spm

2 http://www.restfmri.net

3 http://www.fil.ion.ucl.ac.uk/spm
